# Epistemic injustice and mental health research: A pragmatic approach to working with lived experience expertise

**DOI:** 10.3389/fpsyt.2023.1114725

**Published:** 2023-03-28

**Authors:** Celestin Okoroji, Tanya Mackay, Dan Robotham, Davino Beckford, Vanessa Pinfold

**Affiliations:** ^1^Black Thrive Global, London, United Kingdom; ^2^Department of Psychological and Behavioural Science, London School of Economics and Political Science, London, United Kingdom; ^3^The McPin Foundation, London, United Kingdom

**Keywords:** lived experience, epistemic injustice, elite capture, epistemic exploitation, mental health

## Abstract

“Epistemic injustice” refers to how people from marginalized groups are denied opportunities to create knowledge and derive meaning from their experiences. In the mental health field, epistemic injustice occurs in both research and service delivery systems and particularly impacts people from racialized communities. Lived experience involvement and leadership are often proposed as methods of combatting epistemic injustice, a tool for ensuring the views of people at the center of an issue are heard and can inform decision-making. However, this approach is not without challenges. In this paper, we draw on our work as intermediary organizations that center lived experience perspectives to challenge epistemic injustice. We highlight two problems we have identified in working in the mental health research field: “elite capture” and “epistemic exploitation”. We believe that these problems are barriers to the radical and structural change required for epistemic justice to occur. We propose a pragmatic approach to addressing these issues. Based on our work we suggest three considerations for researchers and our own organizations to consider when involving people with lived experience. These include reflecting on the purpose of creating knowledge, with a focus on impact. Embedding lived experience roles, with appropriate employment, support and remuneration, and acknowledging that it may be necessary to work alongside existing systems as a “critical friend” while developing new spaces and structures for alternative forms of knowledge. Finally, the mental health research system needs to change. We believe these three considerations will help us better move toward epistemic justice in mental health research.

## 1. Introduction

“Epistemic injustice” is a form of systemic discrimination relating to the creation of knowledge (1). It occurs when people from marginalized groups are denied capacity as “epistemic agents” (i.e., as creators of knowledge), and are diminished or excluded from the process of creating meaning (2). Such exclusion creates conditions in which the lived experiences of marginalized people are primarily interpreted by people who do not share their social position (3). Consider the example of Cartwright, an early proponent of racial medicine, who observed enslaved Africans escaping from their slave masters. He interpreted this behavior as evidence of a mental illness called “drapetomania”, ignoring more plausible explanations (4). Contemporary examples of such scientific racism are plentiful (5).

Epistemic injustice is common in mental health care and mental health research. Historically, the knowledge of people with “lived experience” of a mental health issue has been devalued in favor of clinical, academic, and professional knowledge (6). Those delivering mental health care, for example, psychiatrists, psychologists and other support roles, are afforded the assumption of credibility within the mental health system and are prioritized in shaping its policies. Concurrently, those receiving mental health care are often less influential, even when shared decisions are made or feedback is sought, despite the stakes being higher for them in terms of outcomes. More broadly, the evidence base of clinical guidelines tends to rely on positivist notions of a research “gold standard” hierarchy, which marginalizes experiential knowledge in favor of systematic reviews, meta-analyses and randomized controlled trials (7, 8).

“Lived experience involvement”, “co-production” and other such terms are underpinned by a recognition of the epistemic power dynamics in the provision of mental health services and knowledge creation. As an approach, lived experience work recognizes that marginalized people are rarely afforded the opportunity to theorize their own experiences and generate solutions. Accordingly, we explore the experiences of two UK third-sector organizations, Black Thrive and the McPin Foundation, that prioritize “lived experience” to combat epistemic injustice. We draw from our work as intermediary organizations to discuss challenges we have identified in moving toward epistemic *justice* in the health and social care infrastructure and research ecosystem. The paper concludes with our perspective on taking a pragmatic approach to achieving progress. It is important to note that there are different views on what constitutes lived experience expertise (9), for the purpose of this paper it refers to the knowledge and skills gained through the experience of a particular issue or set of circumstances rather than academic or professional knowledge. In the context of our work, this often includes the experience of poor mental health and racialization.

## 2. Background

Our ability to acquire knowledge and understanding is influenced by our social positioning, identity, and experiences (10, 11). At the same time, when these characteristics are associated with a marginalized group, the knowledge acquired may be seen as less credible due to bias or prejudice against the group (1, 12). The exclusion of knowledge from marginalized groups creates a conceptual vacuum. This has real-world impacts as policy and practice decisions are being made in the absence of “conceptual resources” based on lived experience knowledge (or “standpoint”).

Feminist standpoint epistemologists argue that knowledge and new conceptual resources can be created through struggle and critical engagement with oppression. These new conceptual resources dependent on, and developed by, marginalized communities expand our understanding of social situations. Consequently, models and theories developed from lived experience perspectives better recognize the complexity of marginalization and may generate tools for more equitable allocation of societal resources. As Toole says “a conceptual resource is developed to fill some gap in our conceptual understanding, but these resources travel only if they are found to be useful by those who are similarly situated” (11).

An example of this process is the rise of service user involvement in mental health research (13) and Mad Studies (14, 15). The knowledge created by psychiatric survivors has been instrumental over the past 25 years in enhancing mental health practice and research (16, 17). For example, both peer support in the community and peer support workers within mental healthcare were implemented based on research underpinned by survivor knowledge and leadership (18–20). Further, a more recent example from our work was research into the disproportionate impact (including mental health impacts) of the COVID-19 pandemic on marginalized communities (21) undertaken by community peer researchers in one borough of London, United Kingdom (22).

Organizations such as Black Thrive and McPin exist in an “intermediary” space between people with lived experience of mental healthcare, academia and/or practice. We may be asked by academic researchers to broker relationships with people with “lived experience’ and to bring “marginalized” perspectives into academic systems. We have spent a combined 15 years championing the value of lived experience expertise, including in leadership positions, and can offer knowledge on the practicalities of doing so. We are seeing more interest in “inviting in” lived experience, with many funders now making it a requirement, however, this comes with the risk of tokenistic involvement. On the surface, people in the knowledge creation ecosystem may be eager (or coerced) to include lived experience. Nevertheless, it exists in the shadow of a positivist medical model of psychiatry which perpetuates epistemic injustice (6, 23).

There are significant barriers to the creation and legitimization of lived experience knowledge and research paradigms led by mental health survivors and service users. Knowledge production is mostly geared toward higher education institutions. Working outside academia, being employed in third-sector organizations or as independent research consultants inhibits the legitimation of lived experience research. In our experience on university-led research projects, lived experience contributors mostly work in *ad-hoc* roles contained in “advisory groups”, with knowledge generation controlled by those in senior positions. Limited funding, short-term contracts and tight deadlines do not allow sufficient resources for training and development for some people with lived experience, and they face challenges within systems that are rigid in relation to academic culture or workplace expectations. These issues also limit the ability of academic teams to learn and invest in approaches such as co-production and community research. Those affiliated with a university-based research team, such as peer researchers using lived experience as part of their role, tend to be part-time and face assimilation pressures to maintain the status quo. In academia, “knowers” are expected to hold certain kinds of conceptual knowledge to navigate the system and follow traditional and mainstream approaches. Breaking away from these normative codes requires extensive efforts, including emotional labor (24) and there are challenges when bringing lived experience into existing, often harmful, structures.

## 3. Our concerns

We know there are challenges in achieving epistemic justice. We identify two mechanisms, “elite capture” (25) and “epistemic exploitation” (11), which limit the impact of lived experience in improving research to genuinely reflect, and be led by, the needs and experiences of people at the center of an issue.

### 3.1. Elite capture

Roles like “lived experience consultant” or “lived experience researcher” are subject to selection pressures. Social advantages determine who engages and excels in such positions. This elevation and maintenance of a small cohort of marginalized people can be considered “elite capture” (25). In mental health, lived experience involvement is reliant on normative conceptions of mental wellness. Those deemed “too unwell” (e.g., sectioned), “too mad” (e.g., holding alternative views), or “unskilled” (e.g., unable to communicate in ways valued by the system) are excluded. Those invited to contribute are those who can work within systemic constraints of academic and professional behavioral norms, forming an elite subset of a marginalized group. Similarly, the deference of powerful actors to a small “lived experience elite” may buttress the current system, leading to fewer voices, less diversity, narrower goals, and greater alignment between the “lived experience” and elite interests. This reduces the potential for radical solutions.

Nevertheless, we recognize that the experience of trauma *per se* is not adequate preparation for contributing lived experience to knowledge production. Other expertise, skills and resilience are also required. Having an “elite “group who can manage the burdens of lived experience involvement allows *some* influence in the structure of academia. These people can use their lived experience to gain insider knowledge of systems, enabling them to potentially advocate for more voices, translate jargon for others and to move into substantive (non-precarious) roles. In time, this may contribute to systemic change by embedding marginalized voices, but this alone will not lead to epistemic justice because it also relies on gaining patronage and approval from those in existing positions of power or privilege.

### 3.2. Epistemic exploitation

Epistemic exploitation is an epistemic injustice that arises from the expectation of dominant groups that marginalized people will educate them through testimony as to their oppression (11). Indeed, there are many cases in which lived experience is essentialized (26) and isolated from other knowledge and expertise. The “isolation” of lived experience expertise from other forms of expertise prevents people from providing other kinds of insight. It can lead to lived experience expertise being reduced to a testimony (epistemic exploitation), of limited influence and usefulness, distracting from the overall goal of equality and justice. As Lorde said,

“This is an old and primary tool of all oppressors to keep the oppressed occupied with the master’s concerns. Now we hear that it is the task of women of color to educate white women–in the face of tremendous resistance–as to our existence, our differences, our relative roles in our joint survival. This is a diversion of energies and a tragic repetition of racist patriarchal thought.” (27)

Epistemic exploitation reinforces power imbalances. Those in power may fear their own authority and privileges (gained through study and professional experience) being undermined or devalued. They may worry about getting the “right” people involved, or for the need for “representative” people, creating barriers for marginalized people, including Black people and/or people who have experienced mental distress, who are more likely to have had their journeys in education and employment disrupted. Additional challenges arise when asking people to center their lived experience of systems and services that may have harmed them, such as a Local Authority or the National Health Service (NHS), particularly when those systems are unlikely to change.

Our organizations have recently worked together to address systemic change during an employment project based in south London. In the Black Thrive Employment Programme (evaluated by McPin), Black people with experience of living, or caring for someone, with a long-term health condition were part of a “working group”. The group used their collective experience to make funding decisions about local employment support, redistributing power away from funders to local people. Peer researchers were recruited to deliver a developmental evaluation of the program and join its working group. The program created accessible resources, developed from working group discussions and decision making, i.e., “knowledge developed by knowers”.

The working group is an example of where we have experienced elite capture. Members were selected as representatives of local people racialized as Black and with long-term health conditions. This group reflected on representing the “Black experience”, a concept that they felt was homogenizing. The group also lost members over time. The remaining group was more effective at meeting project demands, which included making funding decisions in a way that replicated funder practices. At the same time, and possibly as a result, group members reported feeling pressured to make the “right decisions” (i.e., the ones that traditional funders would make). We sought to remedy this by recognizing the diversity of opinion and individual experiences, providing training, and concluding that the group could not be representative of the wider population and needed better terms of reference. Of interest, this group were engaged from conception in using a range of both lived experience and other skills, including graphic design, facilitation and networking, consequently limiting the impacts of epistemic exploitation.

## 4. Progressing epistemic justice

In practice, Black Thrive and McPin conduct, and support involvement in, research. But we also act as “critical friends”, challenging academic orthodoxy and supporting people with lived experience to influence or to do their own research, including as embedded peer researchers. There are unintended consequences of this; we may contribute to elite capture or epistemic exploitation. As intermediary organizations with limited power in academic systems, we struggle to effect sustainable change through incremental involvement and increased recognition of lived experience expertise. However, through our experience of working in this way, we see three issues that academics and other researchers, including ourselves, should consider.

First, researchers must consider what the purpose of knowledge is. Research that includes lived experience should take a pragmatic approach to knowledge, considering it as a tool for action “which brings us into a more or less satisfactory relation with the world” (28). Pragmatism is an orientation which evaluates research in relation to the outcomes and other consequences it generates. It invites us to consider not “how” research is done but its effects. Incorporating lived experience into a research project does not guarantee that the consequences of the research are epistemically just or even have the potential to create justice. Researchers and organizations should reflect on whether the knowledge we create serves any useful purpose for those with lived experience. If social change is not a core aim of the research, then inviting lived experience into the research process can be oppressive in its own right.

Our understanding of who the knowledge serves is as important as the knowledge itself (28). We may find ourselves balancing lived experience against learned (or professional) experience alongside academic expertise, drawing on the strengths of all positions and highlighting their value to have real-world impact. At Black Thrive and McPin, we care about what new knowledge is intended to do, whose interests are served, and what its wider impacts may be (positive or negative), keeping in mind unintended consequences that may perpetuate epistemic injustice.

Second, a good proxy for the value placed on lived experience are conditions of employment and remuneration. Research organizations and funders must ensure more people with lived experience define the focus of research and occupy meaningful roles to make decisions. In many instances, academics and other professionals will argue that lived experience is central to their research while at the same time remunerating lived experience via temporary payroll arrangements and one-off payments. This casualization maintains a status quo where people in senior positions have more power to influence knowledge creation and limits the pool of people who can work in such precarious employment relationships. In this regard, Black Thrive and McPin operate differently, because we recognize the importance of life experience in understanding and changing systems of oppression and marginalization. We specifically recruit people with those experiences into substantive roles. In both organizations, most substantive staff have lived experience of mental health issues, caring responsibilities or, in the case of Black Thrive – anti-Black racism. We also have *ad-hoc* positions based on people’s preferences to allow accessibility for those who prefer fewer hours or greater flexibility. If lived experience is central to research, then it should be reflected in the workforce and in our research methods. Driven by neoliberalism structural workforce issues favor consultancy models and commodify trauma and oppression, in turn limiting the radical potential of lived experience.

Third, working pragmatically may mean working with unjust systems rather than overhauling them. This can conflict with the political, activist roots of working from a lived experience perspective (9, 14, 15, 29). The irony is not lost on us. Radical change calls for the creation of “new rooms” (30) where hierarchies are flattened and diverse experiences are heard, rather than bringing lived experience into the same structures that created the current problems (6, 25). We have found new spaces and structures do not always accommodate the needs of marginalized groups within marginalized groups because of elite capture. A positive example of a “new room” is the recently launched International Mad Studies Journal, seeking to create spaces for alternative knowledge and marginalized voices, and operating outside the traditional academic publishing system (31). In working toward creating epistemic justice, we must reflect on the voices we hear, and more importantly, those we do not hear.

## 5. Conclusion

Epistemic injustice is prevalent across mental health care and research because the expertise of people with lived experience is devalued in favor of “professional” knowledge. Organizations such as Black Thrive and McPin operate between lived experience and academia. In this paper, we have attempted to share our experiences of some of the ways in which the potential impact of lived experience is diminished by wider research culture.

We highlighted the problem of elite capture, the idea that those who are able (and invited) to contribute lived experience may be unrepresentative of those who share the experience. Those who are brought in to contribute lived experience may become, or are selected based on, their alignment with the status quo. We also highlighted the issue of epistemic exploitation, long recognized by Black feminists (11, 27), where lived experience becomes a perpetual testimony with little influence or utility for justice.

Our observations remind us that incorporating lived experience expertise into dominant academic paradigms will not create systemic change. We proposed the following principles to help researchers and others progress epistemic justice. The first is for greater pragmatism when considering what the research will do and how it can potentially bring about social justice. Many people are tired of engaging with research that does not lead to change particularly when it is used to maintain the status quo. Secondly, researchers and their institutions must consider the terms under which they employ people with lived experience expertise. If lived experience is crucial then it must be remunerated with stable conditions of employment. The current “consultancy” model only creates space for a tiny minority who can afford such precarity. Finally, we believe that people with lived experience must be supported to pursue their own research priorities, not just to support the interests of others. This requires new spaces, not recreating the same structures that produced the current conditions.

## Data availability statement

The original contributions presented in the study are included in the article/supplementary material, further inquiries can be directed to the corresponding author.

## Author contributions

CO, TM, VP, DR, and DB contributed to the conception and design of the manuscript. CO wrote the first draft of the manuscript. TM and DR wrote the second draft of the manuscript, VP and DB wrote sections of the manuscript. All authors contributed to manuscript revision, read, and approved the submitted version.

## Funding

Funding for time spent on this manuscript was received from Impact on Urban Health, Lankelly Chase and Black Thrive.

## Conflict of interest

The authors declare that the research was conducted in the absence of any commercial or financial relationships that could be construed as a potential conflict of interest.

## Publisher’s note

All claims expressed in this article are solely those of the authors and do not necessarily represent those of their affiliated organizations, or those of the publisher, the editors and the reviewers. Any product that may be evaluated in this article, or claim that may be made by its manufacturer, is not guaranteed or endorsed by the publisher.
